# Practice of Hardening and Tempering Steel

**Published:** 1862-02

**Authors:** 


### Selections.

PRACTICE OF HARDENING AND TEMPERING STEEL.—When in the fire the steel becomes altogether expanded, and in the water its outer crust is suddenly arrested, but with a tendency to contract from the loss of heat, which can not so rapidly occur at the central part; it may be, therefore, presumed that the inner bulk continues to contract after the outer crust is fixed, and which tends to tear the two asunder, the more especially if there be any defective part in the steel itself. An external flake, of greater or less extent, not unfrequently shells off in hardening; and it often happens that works remain unbroken for hours after removal from the water, but eventually give way and crack with a loud report, from the rigid, unequal tension produced by the violence of the process of hardening.

The contiguity of thick and thin parts is also highly dangerous, as they can neither receive nor yield up heat, in the same times; the mischief is sometimes lessened by binding pieces of metal around the thin parts with wire, to save them from the action of the cooling medium. Sharp, angular notches are also fertile sources of mischief, and, where practicable, they should be rejected in favor of curved lines.

As regards both cracks and distortions, it may, perhaps, be generally said that their avoidance depends principally upon *manipulation, or the successful management of every step;* first, the original manufacture of the steel, its being forged and wrought, so that it may be equally condensed on all sides with the hammer, otherwise, when the cohesion of the mass is lessened from its becoming red hot, it recovers in part from any unequal state of density in which it may have been placed.

While red hot, it is also in its weakest condition; it is therefore prone to injury, either from incautious handling with the tongs, or from meeting the sudden cooling action irregularly, and, therefore, it is generally best to plunge works vertically, as all parts are then exposed to equal circumstances, and less disturbance is risked than when the objects are immersed obliquely or sideways into the water; although, for swords, and objects of similar form, it is found the best to dip them exactly as in making a vertical downward cut with a sabre, which, for this weapon, is its strongest direction.

Occasionally objects are clamped between stubborn pieces of metal, as soft iron or copper, during their passage through the fire and water. Such plans can be seldom adopted, and are rarely followed, the success of the process being mostly allowed to depend exclusively upon good general management.*

*In all cases* the thick, unequal scale left from the forge should be ground off before hardening, in order to expose a clean metallic surface, otherwise the cooling medium can not produce its due and equal effect throughout the instrument. The edges also should be left thick, that they may not be burned in the fire; thus it will frequently happen that the extreme end or edge of a tool is inferior in quality to the part within, and that the instrument is much better after it has been a few times ground; in urging this point, I can not do better than quote the couplet inserted in Moxon's 'Mechanic Exercises,' and which he describes as having been very old and familiar to smiths even in his day—

" He that will a good Edge win,
Must Forge thick and Grind thin."

Thirdly, the heat for tempering or letting down. Between the extreme conditions of hard and soft steel there are many intermediate grades, the common index for which is the oxidation of the brightened surface, and it is quite sufficient for practice. These hints, and their respective approximate temperatures, were tabulated by Mr. Stodart.

| | Degrees. | |
|---|---|---|
| 1. Very pale straw yellow | 430 | Tools for metal. |
| 2. A shade of darker yellow | 450 | |
| 3. Darker straw yellow | 470 | Tools for wood & screw tops, etc. |
| 4. Still darker straw yellow | 490 | |
| 5. A brown yellow | 500 | |

* Mr. Jordan informs me that in making the magnets for Fox's dipping needles, which are about ten inches long, one fourth of an inch wide, and the two hundredth part of an inch thick, this precaution entirely failed; and the needles assumed all sorts of distortions when released from between the stiff bars within which they were hardened. The plan was eventually abandoned, and the magnets were heated in the ordinary way within an iron tube, and were set straight with the hammer, after being let down to a deep orange or brown color. Steel, however, is in the best condition for the formation of good permanent magnets when perfectly hard.

| | | | |
|---|---|---|---|
| 6. | A yellow, tinged slightly with purple | 520 | Hatchets, chipping chisels, and other percussive tools, saws, etc. |
| 7. | Light purple | 530 | |
| 8. | Dark purple | 550 | Springs. |
| 9. | Dark blue | 570 | |
| 10. | Paler blue | 600 | Too soft for the above purposes. |
| 11. | Still paler blue | 610 | |
| 12. | Still paler blue, with a tinge of green | 630 | |

The first tint arrives at about 430° F., but it is only seen by comparison with a piece of steel not heated; the tempering colors differ slightly with the various qualities of steel.*

The heat for tempering being moderate, it is often supplied by the part of the tool not requiring to be hardened, and which is not therefore cooled in the water. The workman first hastily tries with a file whether the work is hard; he then partially brightens it at a few points with a piece of grindstone or an emery stick, that he may be enabled to watch for the required color; which attained, the work is usually cooled in any convenient manner, lest the body of the tool should continue to supply heat. But when, on the contrary, the color does not otherwise appear, partial recurrence is had to the mode in which the work was heated, as the flame of the candle, or the surface of the clear fire, applied, if possible, a little below the part where the color is to be observed, that it may not be soiled by the smoke.

A very convenient and general manner of tempering small objects is to heat to redness a few inches of the end of a flat bar of iron, about two feet long; it is laid across the anvil, or fixed by its cold extremity in the vice; and the work is placed on that part of its surface which is found by trial to be of the suitable temperature, by gradually sliding the work toward the heated extremity. In this manner many tools may be tempered at once, those at the hot part being pushed off into a vessel of water or oil, as they severally show the required color; but it requires dexterity and quickness in thus managing many pieces.

Vessels containing oil or fusible alloys carefully heated to the required temperatures have also been used, and I shall

* The knife edges, for Captain Kater's experimental pendulum, were very carefully hardened and tempered in a bath heated to 430°; being then found too soft, they were rehardened and tempered, at only the heat of boiling water, after which they were considered admirably suited to their purpose.

have to describe a method called "*blasting off*," resorted to for many articles, such as springs and saws, by heating them over the naked fire until the oil, wax, or composition in which they have been hardened ignites; this can only occur when they respectively reach their boiling temperature, and are evaporated in the gaseous form.

The period of letting down the works is also commonly chosen for correcting, by means of the hammer, those distortions which so commonly occur in hardening; this is done upon the anvil, either with the thin pane of an ordinary hammer, or else with a *hackhammer*, a tool terminating at each end in an obtuse chisel edge, which requires continual repair on the grindstone.

The blows are given on the hollow side of the work, and at right angles to the length of the curve; they elongate the concave side, and gradually restore it to a plane surface, when the blows are distributed consistently with the positions of the erroneous parts. The hack-hammer unavoidably injures the surface of the work, but the blows should not be violent, as they are then also more prone to break the work, the liability to which is materially lessened when it is kept at or near the tempering heat, and the edge of the hack-hammer is slightly rounded.—*British Journal of Dental Science.*

